# Accurate and stable equal-pressure measurements of water vapor transmission rate reaching the 10^−6^ g m^−2^ day^−1^ range

**DOI:** 10.1038/srep35408

**Published:** 2016-10-17

**Authors:** Yoichiro Nakano, Takashi Yanase, Taro Nagahama, Hajime Yoshida, Toshihiro Shimada

**Affiliations:** 1 Division of Applied Chemistry, Faculty of Engineering, Hokkaido University, Kita 13 Nishi 8, Kita-ku, Sapporo, 060-8628, Japan; 2 The National Metrology Institute of Japan, The National Institute of Advanced Industrial Science and Technology, 1-1-1 Umezono, Tsukuba, 305-8560, Japan

## Abstract

The water vapor transmission rate (WVTR) of a gas barrier coating is a critically important parameter for flexible organic device packaging, but its accurate measurement without mechanical stress to ultrathin films has been a significant challenge in instrumental analysis. At the current stage, no reliable results have been reported in the range of 10^−6^ g m^−2^ day^−1^ that is required for organic light emitting diodes (OLEDs). In this article, we describe a solution for this difficult, but important measurement, involving enhanced sensitivity by a cold trap, stabilized temperature system, pumped sealing and calibration by a standard conductance element.

Mechanically flexible displays and other electronic circuits can be fabricated from organic semiconductors on polymer film substrates^1^,^2^,^3^,^4^. One of the greatest obstacles for the mass production of these devices is the barrier coatings that can block the water vapor transmission to the organic molecules and electrodes. The required water vapor transmission rate (WVTR) is in the range of 10^−6^ g m^−2^ day^−1 ^5. Although some instruments to measure the WVTR values are commercially available, the actual measurement for ultrathin films is difficult and the reliable sensitivity limit does not reach this range. The *de facto* standard of the WVTR measurement is evaluating the corrosion of an encapsulated calcium metal thin film^6^,^7^,^8^ (called the “Ca method”), but it requires very a long time ranging from weeks to months. The main reason is that the amount of water involved is very low. Assuming the sample area of 60 cm^2^ (~90 mm diameter) with a WVTR value of 1 × 10^−6^ g m^−2^ day^−1^, the amount of water transmitted per hour is 2.5 × 10^−10^ g.

The measurement scheme common to most of the existing instruments^9^, except for the Ca method is illustrated in Fig. 1. The transmitted water molecules are usually introduced in a chamber with a vacuum pump or a continuous flow. The WVTR value is estimated from the concentration of water in the chamber at the steady state. When a vacuum pump is used, the detection can be done rather easily with a vacuum gauge^10^ (2.5 × 10^−10^ g H_2_O in 100 cm^3^ at RT corresponds to 3 × 10^−4^ Pa) or other instruments because only H_2_O molecules exist in the detector. However, a serious problem is the pressure difference that will exist between the two faces of the barrier film sample, which might damage the sample and coatings if the film is very thin and flexible. Various equal-pressure techniques have been proposed to avoid this problem. They use a continuous flow of atmospheric carrier gas in the “detector side” or “dry side”. The problem is that the detection requires a very sensitive technique specific to H_2_O, because the atmospheric pressure carrier gas exists in the detector. The H_2_O concentration in the carrier gas is of part-per-billion(ppb) level. These methods include cavity ring down spectroscopy^11^ and other multipath optical absorptions, or mass spectroscopy with an ionization sensitizer operated with differential pumping (“API-MS”)^9^. It is common knowledge among the users that it requires a very long time for stabilization of the instruments. A value lower than 1 × 10^−5^ g m^−2^ day^−1^ is accepted as ambiguous, independent of whether the measurement is done by equal-pressure or pressure-difference methods. Actually, no detailed measurement can be found in the literature in the range of 10^−6^ g m^−2^ day^−1^, except for the Ca method or extrapolated estimations by accelerated experiments at elevated temperatures^12^.

The reason might be simple. A problem that has not been previously mentioned in the literature is residual water molecules adsorbed on the chamber wall. Suppose the surface area of the chamber is 100 cm^2^. The amount of water on the surface with a monolayer absorption is experimentally evaluated 3.0 × 10^14^ molecules/cm^2^, *i.e.* 3 × 10^16^ molecules or 9 × 10^−7^ g on 100 cm^2^ surface, which is 3600 times greater than the amount transmitted from the 10^−6^ g m^−2^ day^−1^ sample (60 cm^2^) per hour. The amount of the adsorbed water must be stable at the steady state within the range of 1/3600. If this value deviates, the obtained WVTR value fluctuates and the measurement will be impossible. The adsorption enthalpy of H_2_O molecules on electropolished stainless steel is *ΔH* ~ 0.10 eV^13^. The allowance of the temperature deviation *ΔT* is estimated from *f*(*T*+*ΔT*)/*f*(*T*) = 1–1/3600, where *f*(*T*) = exp(-*ΔH*/*k*_B_*T*), which results in a *ΔT* ~ 20 mK when *T* = 300 K. The surface area of the instrument inner wall including the pump and detector is usually greater than 100 cm^2^, which makes the allowance of *ΔT* worse. Deviation in the room temperature and other environments (*e.g.*, sunshine, wind from air conditioner) will easily exceed this limit.

Another problem is calibration of the detector. The detectors sensitive enough for the measurement of the 10^−6^ WVTR range require calibration. We have noticed that the pumping speed is also a variable, if a vacuum pump is used in the instrument. It is less recognized that the sensitivity of electron multiplier used in the mass spectrometer fluctuates by more than 200%.

In this paper, we describe our new instrument equipped with a calibration device and highly sensitive amplified measurement scheme with a cold trap^14^,^15^. The important instrumental know-how, such as the calibration procedure, prevention of the water vapor entering through the seal, and data analysis, will be presented. We demonstrate the actual measurement of barrier samples having a 1.7 × 10^−5^ g m^−2^ day^−1^ WVTR. The standard deviation was 7 × 10^−6^ g m^−2^ day^−1^ over repeated measurements for one year, which has never been reported with full analysis.

## WVTR Measurement

**Figure 2:**
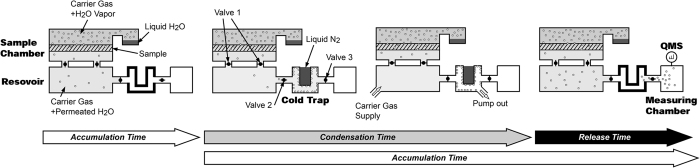
Measurement procedure using cold trap amplification.

The measuring system mainly consists of a sample chamber, which is equipped with a water container, a reservoir, a cold trap, and a measuring chamber, which is equipped with a quadruple mass spectrometer (QMS) (Fig. 2). The detailed process of measurement is described in the Supplementary Information, but briefly explained in this section.

A film sample with a Φ60 mm circle permeated area, was placed in the sample chamber, then held down by a fluoropolymer o-ring from both sides. The sample chamber has a outer hull to pump out. This is necessary to prevent the water vapor from the wet side or in the atmosphere to diffuse into the dry side through the o-ring. An ordinary elastomer o-ring is highly permeable to H_2_O compared to the barrier sample. By pumping the outer hull, the H_2_O molecules diffused from the wet side will be pumped out and does not permeate through the o-ring seal of the dry side. The whole system, inside and outside of the sample chamber was pumped out and heated (80–130 °C) to remove the water molecules in the sample and on the chamber wall. After 24 hours, the chamber containing the sample was cooled to the measurement temperature (40 °C).

The carrier gas with an H_2_O concentration less than 1 ppb, produced by passing through an alkali getter (GP-10, Pureron Japan, Inc.), was supplied to the wet and dry sides of the system until it reached atmospheric pressure. The o-ring seal was mechanically pressed from the both sides and water vapor container was connected to the wet side. In order to keep a specific humidity in the upper side, the temperatures of liquid water in the water container and carrier gas were separately controlled (two-temperature method).

The H_2_O vapor permeated through the sample was quantified by amplification using a cold trap. After the accumulation time (3 hours), the dry side of the sample space was disconnected from the downstream to prevent a pressure deviation and connected to the cold trap. The pressures of the wet and dry sides are regulated by two independent pressure gauges (0.1% accuracy of 10^5^ Pa) on the both sides and automatic carrier gas supply to the dry side. The temperature of the cold trap was decreased to 77 K to trap the H_2_O molecules. During this operation, the H_2_O vapor keeps permeating through the sample and is accumulated in the sample space.

When the condensation time (within 15 minutes) passes, the remaining carrier gas in the cold trap was pumped out to a high vacuum in order to enable the accurate measurement by a quadrupole mass spectrometer (QMS), while H_2_O molecules are still condensed on inner surface of the cold trap. The cold trap is then quickly heated to 100 °C to release the trapped water, which is detected by the QMS using a stable faraday cup detector.

By using this method, the QMS signal is enhanced by the factor of accumulation time/release time of *c.a.* 20, enabling the detection using the faraday cup of the mass spectrometer also, that is more stable than a secondary electron multiplier. The data points are intermittently obtained (every accumulation time of three hours) and the statistical analysis is easier.

It is noted that after the whole system reaches the steady state, the effect of the dead volume (*e.g.* a part of the dry side of the sample chamber) and the absorption on the inner wall should become negligible, because the amount of water not going to the detector in one cycle will be detected in average, due to the definition of the steady state. The key factor is the stability of the steady state, which is mainly determined by the temperature stability as discussed above.

### Calibration of QMS

The sensitivity of the QMS is not stable for a long period, and shows a tens of percent deviation within a year even when operated using the Faraday cup detector. In this study, calibration is conducted using a reliable leak which was developed by The National Metrology Institute of Japan (NMIJ) of National Institute of Advanced Industrial Science and Technology (AIST) and called the standard conductance element (SCE) for *in situ* calibration^16^. The SCE is made of a stainless-steel sintered filter with the pore size of less than 1 μm. Gas flow through the SCE satisfied the molecular flow condition at pressures up to 10^4^ Pa. The flow rate can be precisely controlled by adjusting the pressure at the upstream side. Note that the flow rate sensitivity is a parameter involving the sensitivity of the QMS and the pumping speed of the system.

For calibration, the flow rate sensitivity *S*_F_ [A s g^−1^] was defined as the QMS ion current increase *ΔI* [A] when the H_2_O vapor was introduced at the flow rate of *F*_W_ [g s^−1^], where the ion current of the QMS (*m/z* = 18) increases from *I*_0_ to *I*_0_ + *ΔI*. The procedure of measuring *S*_F_ is described as follows.

**Figure 3:**
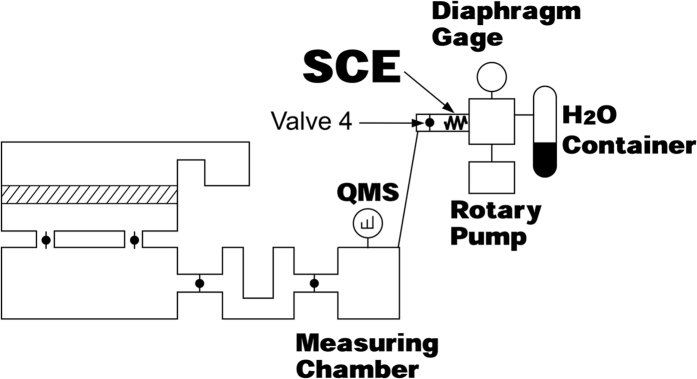
Calibration of QMS sensitivity and pumping speed by SCE.

The SCE with a container filled with water vapor is first attached to the measurement chamber (Fig. 3). “Valve 4” is then opened and the water vapor flows from the container to the chamber. The flow rate *Q*_W_ [Pa∙m^3^ s^−1^] was adjusted by changing the pressure *P*_c_ [Pa] in the container, which is measured by a diaphragm gauge. *Q*_W_ is determined by multiplying *P*_c_ [Pa] and the conductance of the SCE *C*_S_ [m^3^ s^−1^].


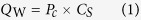


For the WVTR measurement, it is convenient to convert the gas flow rate *Q*_W_ [Pa∙m^3^ s^−1^] to *F*_W_ [g s^−1^].


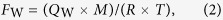


where *M* [g mol^−1^] is molar mass of water, *R* is the gas constant, and *T* [K] is the absolute temperature.

Finally, the flow rate sensitivity *S*_F_ [A∙s g^−1^] is obtained by dividing *ΔI* by *F*_W_.


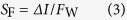


The flow rate sensitivity is measured once a month, and shows some fluctuation ranging from 1.5 to 2.0 × 10^−3^ A∙s g^−1^ during the period of one year.

### Evaluation of detected water

**Figure 4:**
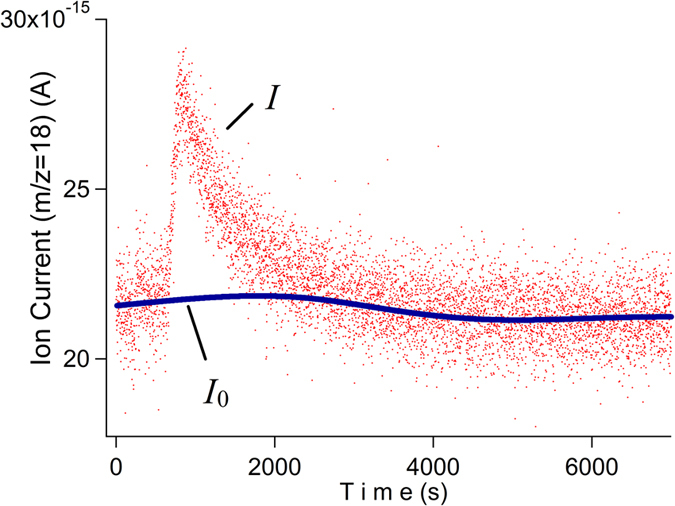
Typical QMS output during heating the cold trap. The solid line *I*_0_ was determined by a spline interpolation of predesignated region out of the peak.

The quantity of permeated water vapor during the accumulation time can be obtained using the QMS signals and flow rate sensitivity. Figure 4 shows an example of the QMS signals during the release of captured water from the cold trap. The difference between QMS ion current *I* and its background *I*_0_ was initially integrated. Since the background slowly fluctuates during the measurement, spline fitting is used for estimating the background *I*_0_. This removal of the slow fluctuation is another merit of using this intermittent measurement scheme, which makes the measurement less sensitive to slow temperature fluctuations of the environment.

To obtain the amount of water *m* [g], the integration was divided by the flow rate sensitivity *S*_F_.


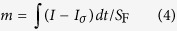


Finally, WVTR [g m^−2^ day^−1^] was obtained as follows


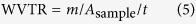


where *A*_sample_ [m^2^] is the sample area, and *t* [day] is the accumulation time. All the data below are shown after this procedure.

### Evaluation of the entire detection system using SCE in place of the sample chamber

**Figure 5:**
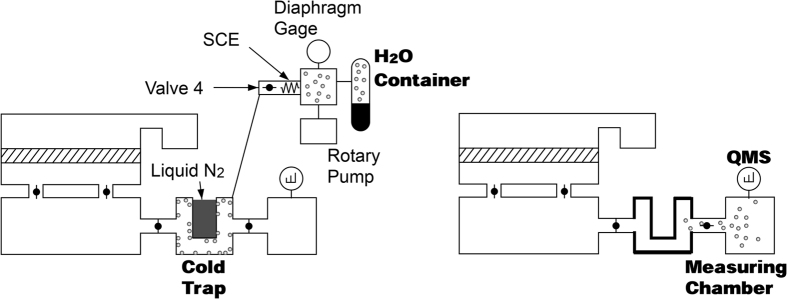
Evaluation of detection system by introducing given amount of water vapor by SCE.

The current problem of the WVTR measurement is that there is no standard film for a reliable and stable WVTR down to the 10^−6^ gm^−2^ day^−1^ range. We used the SCE to evaluate the accuracy of the measurement by connecting it to the detecting system (including cold trap) in place of the sample chamber. As illustrated in Fig. 5, the SCE with a water vapor container is attached to the cold trap. The supply rate of H_2_O was adjusted by changing the pressure in the container. By regulating the time of the connection, the number of the water molecules can be precisely controlled. The cold trap was cooled and heated with the valve operation just as in the WVTR measurement previously described. The details of this experiment are described in the Supplement Information. We note that the calibration and evaluation by SCE can be only partially validated because the sample chamber is bypassed. Although these procedures should be done with a standard high barrier sample or its equivalent, there are no reliable and durable high barrier samples available. We believe that the present procedure is one of the best at present stage.

## Results and Discussion

### Detection system evaluation by SCE

**Figure 6:**
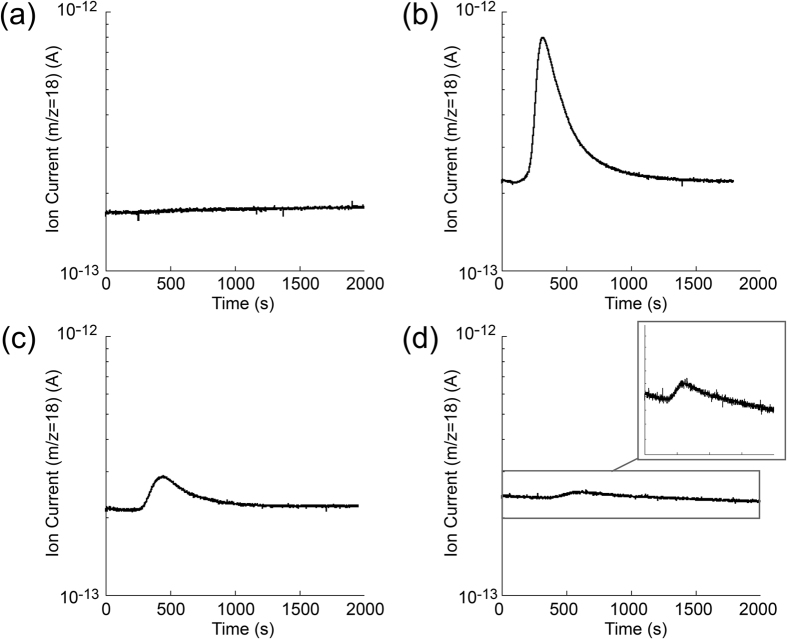
QMS output of cold trap evaluation by SCE. (**a**) #1 in Table 1 (no H_2_O supply) (**b**) #3 (H_2_O supply corresponding to 1.8 × 10^−4^ g m^−2^ day^−1^) (**c**) #5 (1.8 × 10^−5^ g m^−2^ day^−1^) (**d**) #8 (2.0 × 10^−6^ g m^−2^ day^−1^).

Figure 6 shows the QMS signals (m/z = 18) during heating the cold trap for the evaluation experiment. The background levels ranged from 2 to 2.4 × 10^−13^ A which approximately corresponded to 10^−6^ Pa. Without supplying water vapor through the SCE, H_2_O was not detected by the QMS (Fig. 6(a)). The experiments were then carried out by supplying water corresponding to the WVTR 10^−3^ to 10^−6^ g m^−2^ day^−1^. As can be seen in Fig. 6(b–d), 200 seconds after starting the heating of the trap, the ion current (m/z = 18) apparently begins to rise and subsequently fall to the background level within 1500 seconds. Most of captured water was released between 250 and 850 seconds (10 minutes). Figure 6(d) indicates that the water vapor corresponding to lower than 10^−6^ g m^−2^ day^−1^ is sufficiently detectable. These results revealed that the present setup is sensitive enough to detect an extremely small quantity of water corresponding to 10^−6^ g m^−2^ day^−1^. As seen in Fig. 6(d), the peak height of the QMS signal (*m/z* = 18) from its background (2.4 × 10^−13^ A) was 1 × 10^−14^ A. The WVTR of 1 × 10^−6^ g m^−2^ day^−1^ is considered to be near the detection limit in the current configuration, because the peak height of the QMS signal is not very high compared to its background. Since the WVTR is roughly proportional to the peak height from the background, the peak height corresponding to the WVTR of 10^−7^ g m^−2^ day^−1^ is expected to be 10^−15^ A, thereby in order to detect the WVTR 10^−7^ g m^−2^ day^−1^, it is desirable to lower the background below 10^−13^ A.

Table 1 shows the results of the detected water amount as a function of the supplied water amount. The detected values for 1.8 × 10^−4^ g m^−2^ day^−1^ were in good agreement with the supplied water. On the other hand, the result for low 10^−5^ g m^−2^ day^−1^ level deviates at the range of 1 ~ 2 × 10^−5^ g m^−2^ day^−1^. This is already comparable to the highest accuracy in the literature not using the Ca method. The deviation of the actual barrier film results shown later is smaller than this. We consider this deviation in the SCE experiments is due to the fluctuation of the temperature around the SCE and connecting tubing, which was operated at ambient temperature. The stability was further evaluated by measuring the barrier film samples using the sample chamber, which is temperature-controlled to a 100 mK accuracy.

### WVTR measurements of barrier films

We now describe two examples. One was a low-barrier coated polymer film which showed a barrier property of 9 × 10^−4^ g m^−2^ day^−1^ when evaluated by commercially available instrumentation (Technolox). The other was a high-barrier coated one that could not be evaluated by the other setup. The measurements were carried out at 3-hours intervals at 40 °C and 90% RH.

**Figure 7:**
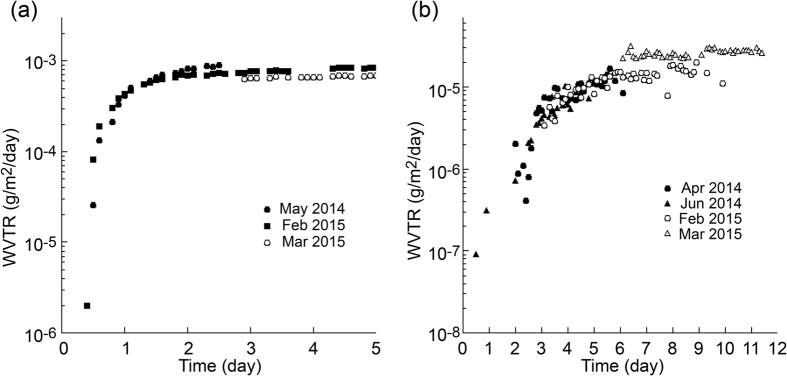
Results of measurement of barrier samples. (**a**) a low barrier (**b**) a high barrier. (**a**) showed 9 × 10^−4^ g m^−2^ day^−1^ WVTR by a commercial instrument, whereas (**b**) was below the detection limit of commercially available instruments.

Figure 7(a) shows the results of the low-barrier coated films. It is clearly seen that WVTR reached a stable value within 3 days. A total of 4 days, including a day for conditioning the sample and the apparatus, were required to obtain the result. The average value of the four separate measurements was 8.3 × 10^−4^ g m^−2^ day^−1^, which is close to the value evaluated by the other setup. The standard deviation of our measurements was 8.1 × 10^−5^ g m^−2^ day^−1^.

Figure 7(b) shows the results of the high-barrier coated films. It took 6 days to reach a rough steady state. A total 7 days are necessary to evaluate these films. The average WVTR value of the four separate measurements was 1.7 × 10^−5^ g m^−2^ day^−1^. The standard deviation of the values after “steady state” was 7.1 × 10^−6^ g m^−2^ day^−1^, which was larger than the standard deviation of repeated measurements over one year.

### Measurement time

Based on the results of the barrier coated polymer films, we discuss the measurement time. Figure 7 shows that the WVTR reached the saturated value within a week (7 days for the 1.7 × 10^−5^ g m^−2^ day^−1^ and 4 days for the 8.1 × 10^−4^ g m^−2^ day^−1^). This saturated time is independent of the detector sensitivity, and dependent on the property of the films. The distribution and density of defects in the thin barrier coating layer (usually < 1 μm thick) is considered as the limiting factor of the water vapor permeation. The steady state permeation was attained within 1–7 days depending on the property of the barrier coating and the diffusion constant of the base polymer.

The ratios of the standard deviations of the averages were estimated to be tens of percent (42% for the high barrier coated films and 10% for the low barrier coated films). One of the causes, which hindered the precise measurements, is the fluctuation of the background H_2_O partial pressure. The fluctuation timescale ranging from minutes to a year was observed, which probably results from the H_2_O adsorption and desorption on the inner surface of the apparatus. Nevertheless, the measurement with a continuous flow or pumping of the permeated water will give the correct value after reaching a steady state. As mentioned in the introduction, the temperature stability is one of the key factors to determine the lower limit of the reliable measurement. Additionally, a change in the barrier strength originating from the sample in the repeated measurement is suspected, which can be caused by a non-uniform distribution of nano-scale defects and larger scale pinholes^17^.

### Error estimation

We have evaluated the possible causes of the error in the present technique. They are (1) sensitivity of the detector including the pumping speed, (2) penetration through the sample seals and (3) adsorption and desorption of water molecules on the inner wall.

(1) was calibrated by SCE in this paper to the accuracy better than + 20%. (2) was minimized by introducing the outer hull that is pumped to high vacuum. The amount of the water vapor escaping from the dry side is negligible if the obvious leak is not present. This can be confirmed by checking the vacuum of the outer hull, which was routinely done in the present experiments. (3) is the most important factor. As already explained in the Introduction section, 1000 cm^2^ stainless steel surface of the inner wall (the present apparatus) will adsorb 12000 times greater than the amount transmitted from a 10^−6^ g m^−2^ day^−1^ sample (60 cm^2^) per 3 hours (the present measurement). The temperature deviation of 100 mK (0.1 K) will make 0.1/300 deviation of adsorbed water. It will make the error corresponding to 4 × 10^−6^ g m^−2^ day^−1^ WVTR, which is reasonably in agreement with the standard deviation (7 × 10^−6 ^g m^−2^ day^−1^) of the data plotted in Fig. 7(b).

### Comparison with other techniques

Although various methods to measure the WVTR have been developed, many of them are used for the purpose of evaluating low barrier films (>10^−4^ g m^−2^ day^−1^), and thereby not suitable for testing OLED application films (10^−6^ g m^−2^ day^−1^). Some methods have recently been proposed to enable the high barrier measurement.

Other techniques, such as cavity ring down spectroscopy and other optical absorption spectroscopies, also suffers from the fluctuation of the adsorbed water in the inner wall system, as well as the differential pressure method. We point out that any method continuously accumulating the water molecules in the system for the increased sensitivity does not reach a steady state. The continuous change in the inner wall adsorption will cause a severe and complex error in the result, unless the inner wall area is very small as in the Ca method. It should be noted that the detector side must be under vacuum for the operation of the SCE, and our cold trap detection scheme is well suited for this calibration method among equal-pressure measurement techniques.

The development of surface coating material for H_2_O molecule adsorption will be essential to further improve the detection limit^18^ as well as improving the temperature stability. Our technique using intermittent detection every several hours is useful for evaluating the accuracy by statistics, and less sensitive to the short period fluctuation of the temperature because the background fluctuation can be removed as shown in Fig. 4.

## Conclusion

We have demonstrated that the WVTR measurement for sub- 10^−5^ g m^−2^ day^−1^ is possible by carefully constructing the measurement system with a sensitive detector such as the QMS amplified by a cold trap. The sensitivity of the entire detection system can be calibrated using a standard conductance element. The information described in this paper will help the development of high barrier films, and as a result, will accelerate the mass production of flexible electronic devices for practical use.

## Experimental

The instrument described here was home-build with specially machined ultrahigh-vacuum compatible components. The temperature of the components were controlled at the optimized temperature for operation with the accuracy of 100 mK. The QMS was an Inficon Transpector 2 that can be at elevated temperatures during the operation. The measurement was automated once the sample was placed in the chamber. The experiments were performed in a room with its temperature controlled by an air conditioner. The barrier-coated films evaluated in Fig. 7 were fabricated by multiple deposition of polymer and inorganic materials.

## Additional Information

**How to cite this article**: Nakano, Y. *et al.* Accurate and stable equal-pressure measurements of water vapor transmission rate reaching the 10^−6^ g m^−2^ day^−1^ range. *Sci. Rep.* **6**, 35408; doi: 10.1038/srep35408 (2016).

## Supplementary Material

Supplementary Information

## Figures and Tables

**Figure 1:**
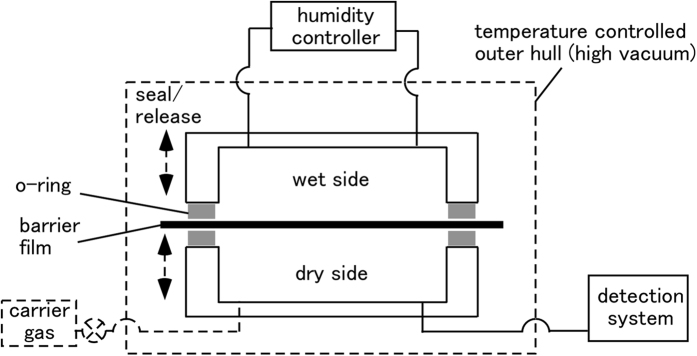
Common components in instrumental measurement of WVTR. Components or operation indicated by dashed line do not always exist.

**Table 1:** Evaluation of the detection system including the cold trap by introduction of given amount of water vapor.

Experiment No.	Supplied water (g m^−2^ day^−1^)	Detected water (g m^−2^ day^−1^)
1	0	not detected
2	1.8 × 10^−4^	2.1 × 10^−4^
3	1.8 × 10^−4^	1.8 × 10^−4^
4	1.8 × 10^−4^	2.2 × 10^−4^
5	1.8 × 10^−5^	3.5 × 10^−5^
6	1.3 × 10^−5^	3.1 × 10^−5^
7	1.3 × 10^−5^	9.8 × 10^−6^
8	2.0 × 10^−6^	6.6 × 10^−6^

The QMS output of underlined experiment No. is shown in Fig. 6.

## References

[b1] Yokota T. *et al.* Ultraflexible organic photonic skin. Sci. Adv. 2, e1501856 (2016). 27152354 10.1126/sciadv.1501856 PMC4846460

[b2] Forrest S. R. The path to ubiquitous and low-cost organic electronic appliances on plastic. Nature 428, 911–918 (2004). 15118718 10.1038/nature02498

[b3] Crone B. *et al.* Large-scale complementary integrated circuits based on organic transistors. Nature 403, 521–523 (2000). 10676955 10.1038/35000530

[b4] Noh Y. Y., Chao N., Caironi M. & Sirringhaus H. Downscaling of self-aligned, all-printed polymer thin-film transistors. Nat. Nanotech. 2, 784–789 (2007). 10.1038/nnano.2007.365 18654432

[b5] Burrows P. E. *et al.* Gas permeation and lifetime tests on polymer-based barrier coatings. Proc. SPIE 4105, 75 (2001).

[b6] Paetzold R., Winnacker A., Henseler D., Cesari V. & Heuser K. Permeation rate measurements by electrical analysis of calcium corrosion. Rev. Sci. Instrum. 74, 5147–5150 (2003).

[b7] Choi J. H. *et al.* Erratum: “Evaluation of gas permeation barrier properties using electrical measurements of calcium degradation”. Rev. Sci. Instrum. 81, 109902 (2010). 10.1063/1.2747168 17614628

[b8] Klumbies H., Müller-Meskamp L., Mönch T., Schubert S. & Leo K. The influence of laterally inhomogeneous corrosion on electrical and optical calcium moisture barrier characterization. Rev. Sci. Instrum. 84, 024103 (2013). 23464228 10.1063/1.4791798

[b9] Suzuki A., Takahagi H., Uehigashi A. & Hara S. Development of reliable technique for evaluating the properties of water vapor barriers. AIP Adv. 5, 117204 (2015).

[b10] Nörenberg H. Miyamoto T., Tsukahara Y. Smith G. D. W. & Briggs G. A. D. Mass spectrometric estimation of gas permeation coefficients for thin polymer membrane. Rev. Sci. Intstum. 70, 2414–2420 (1999).

[b11] Brewer P. J., Goody B. A., Kumar Y. & Milton M. J. Accurate measurements of water vapor transmission through high-performance barrier layers. Rev. Sci. Instrum. 83, 075118 (2012). 22852735 10.1063/1.4738775

[b12] Carcia P. F., McLean R. S., Reilly M. H., Groner M. D. & George S. M. Ca test of Al_2_O_3_ gas diffusion barriers grown by atomic layer deposition on polymers. Appl. Phys. Lett. 89, 031915 (2006).

[b13] Ishihara Y., Kurihara S., Ishihara S., Toda M. & Ohmi T. Trace moisture adsorption onto various stainless steel surfaces - investigation of adsorption heat and adsorption isotherms. J. Surf. Sci. Jpn. 18, 557–563 (1997).

[b14] Shimada T., Takahashi Y. & Kanno T. Highly sensitive and rapid measurement of gas barrier properties of flexible films and sealing resins based on a low temperature trap and mass spectroscopy. Appl. Phys. Exp. 3, 021701 (2010).

[b15] . Nakano Y., Takahashi Y., Kanno T., Yoshida H. & Shimada T. Stable Measurement of 10^−6^ g m^−2^ day^−1^ Water Vapor Transmission Rate in Barrier Materials by Intermittent Accumulation and Release by a Cold Trap, *SID Symposium Digest of Technical Papers,* P-126 (2015).

[b16] Yoshida H., Arai K., Akimichi H. & Kobata T. Newly developed standard conductance element for *in situ* calibration of high vacuum gauges. Measurement 45, 2452–2455 (2012).

[b17] Rossi G. & Nulman M. Effect of local flaws in polymeric permeation reducing barriers. J. Appl. Phys. 74, 5471–5475 (1993).

[b18] Azimi G., Dhiman R., Kwon H.-M., Paxon A. T. & Varanasi K. K. Hydrophobicity of rare-earth oxides. Nat. Mater. 12, 315–320 (2013). 23333998 10.1038/nmat3545

