# Supplementary material for: Accurate and stable equal-pressure measurements of water vapor transmission rate reaching the 10−6 g m−2 day−1 range
Source: Sci Rep. 2016 Oct 17;6:35408. doi: 10.1038/srep35408 (PMC5066313; doi:10.1038/srep35408)
Supplement: Supplementary Information [file srep35408-s1.doc]

**Accurate and rapid equal-pressure measurements of water vapor transmission rate reaching the 10^-6^ g m^-2^day^-1^ range**

Yoichiro Nakano^1^, Takashi Yanase^1^, Taro Nagahama^1^, Hajime Yoshida^2^, Toshihiro Shimada^1^

1. Division of Applied Chemistry, Faculty of Engineering, Hokkaido University, Kita 13 Nishi 8, Kita-ku, Sapporo 060-8628, Japan

2. The National Metrology Institute of Japan, The National Institute of Advanced Industrial Science and Technology, 1-1-1 Umezono, Tsukuba 305-8560, Japan

**I. WVTR measurement procedure**

**
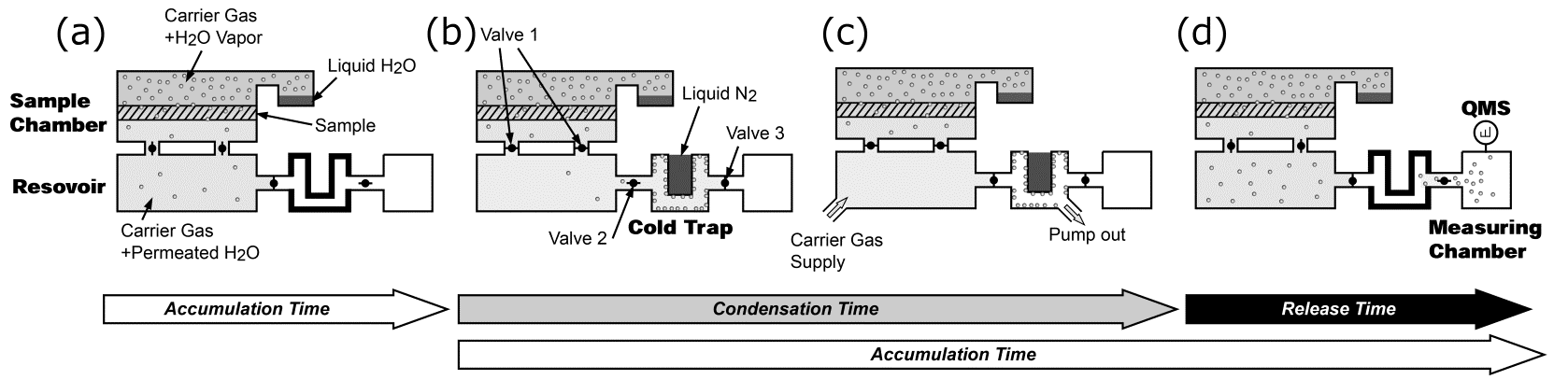
**

Figure S1

A film sample with a Φ60mm circle permeated area, was placed in the sample chamber, then held down by a fluoropolymer o-ring from both sides. The whole system, inside and outside of the sample chamber was pumped out and heated (80-130 °C) to remove the water molecules in the sample and on the chamber wall. After 24 hours, the chamber containing the sample was cooled to the measurement temperature (40°C).

The carrier gas with an H_2_O concentration less than 1 ppb, produced by passing through an alkali getter, was supplied to the wet and dry sides of the system until it reached atmospheric pressure. The o-ring seal was mechanically pressed slowly from the both sides with escaping gas pressures and water vapor container was connected to the wet side. In order to keep a specific humidity in the upper side, the temperatures of liquid water in the water container and carrier gas were separately controlled (two-temperature method). Water vapor permeated through the sample is accumulated in the reservoir for 3 hours (“accumulation time”) (Fig. S1(a)).

The H_2_O vapor permeated through the sample was measured by amplification using a cold trap. After the accumulation time, Valves 1 and 3 were closed and Valve 2 was opened. Then next accumulation time began. At the same time, carrier gas with permeated water began circulating between the reservoir and the cold trap. Immediately after that, the cold trap was cooled by pouring liquid nitrogen, and the inner surface of the cold trap consequently began to adsorb H_2_O molecules (condensation time) (Fig. S1(b). Pressure in the reservoir decreased by approximately 10 % because inside of the cold trap was kept in vacuum before opening the Valve 2. In spite of that, mechanical stress to the sample is avoided because the sample chamber was already disconnected from the reservoir by the Valve 1. During this operation, water vapor kept permeating through the sample and was accumulated in the lower side of the sample space. It should be noted that the isolated volume of the lower side of the sample chamber (less than 10% of the total volume in this experiment) does not cause any error when the steady state is reached after many (a)-(d) cycles are repeated, because the additional water vapor permeated in the accumulation period will be fully detected in the next cycle. When condensation time (15 minutes) passed, the Valve 2 was closed again. Then, the remaining carrier gas in the cold trap was pumped out, while H_2_O molecules were still condensed on inner surface of the cold trap. On the other hand, carrier gas was supplied to the reservoir, which is controlled by the pressure gauges on the both of the dry and the wet sides, until the pressure inside it reaches equal (Fig. S1(c)).

After carrier gas was pumped out from the cold trap, the Valve 3 was opened, and then the cold trap was heated to 100 °C quickly in order to release the trapped water, which is detected by a quadrupole mass spectrometer (QMS) using a faraday cup. The heating was continued for all the detection time but the signal started to appear 200 s after the heating started. The cooling time was 900s (15 minutes), while 300s was used for pumping. The Valve 1 was opened again when the Valve 3 was opened and the permeated water was accumulated in the reservoir (Fig. S1(d)).

Procedures shown in Fig. S1(a) to (d) were repeated until WVTR reached a constant value.

**II. The importance of the steady state**

As stated in the main text, the role of the steady state is very important in the WVTR measurement. The effect of the dead volume (*e.g.* a part of the dry side of the sample chamber) and the absorption on the inner wall should become negligible, because the amount of water not going to the detector in one cycle will be detected in average, due to the definition of the steady state. The key factor is the stability of the steady state, which is mainly determined by the temperature stability.


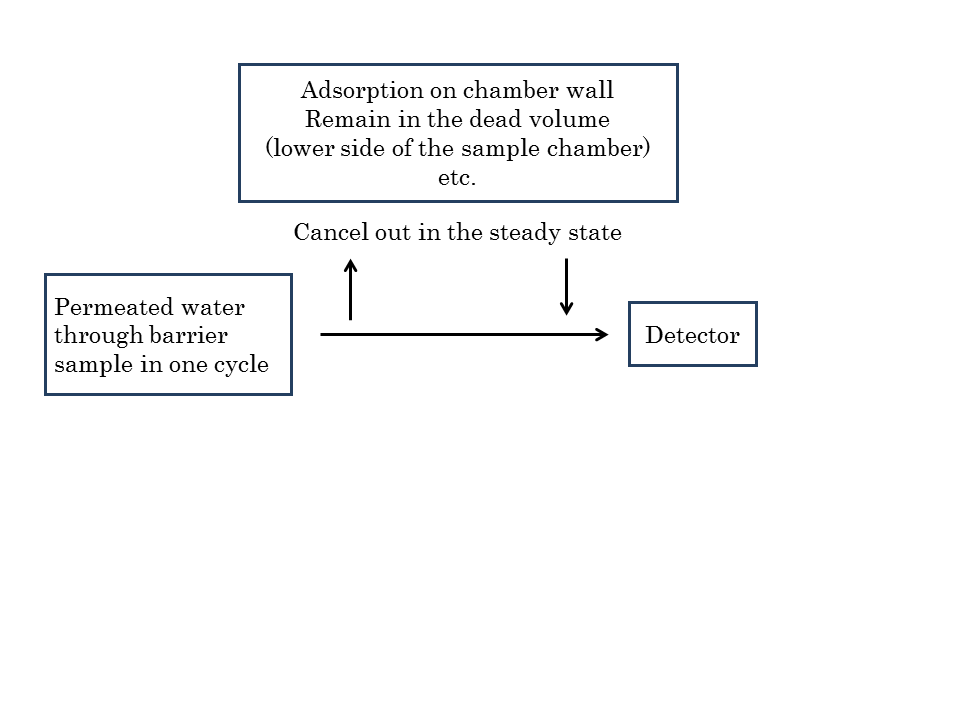


Fig. S2

**III. Procedure for the evaluation of the detection system by the SCE**


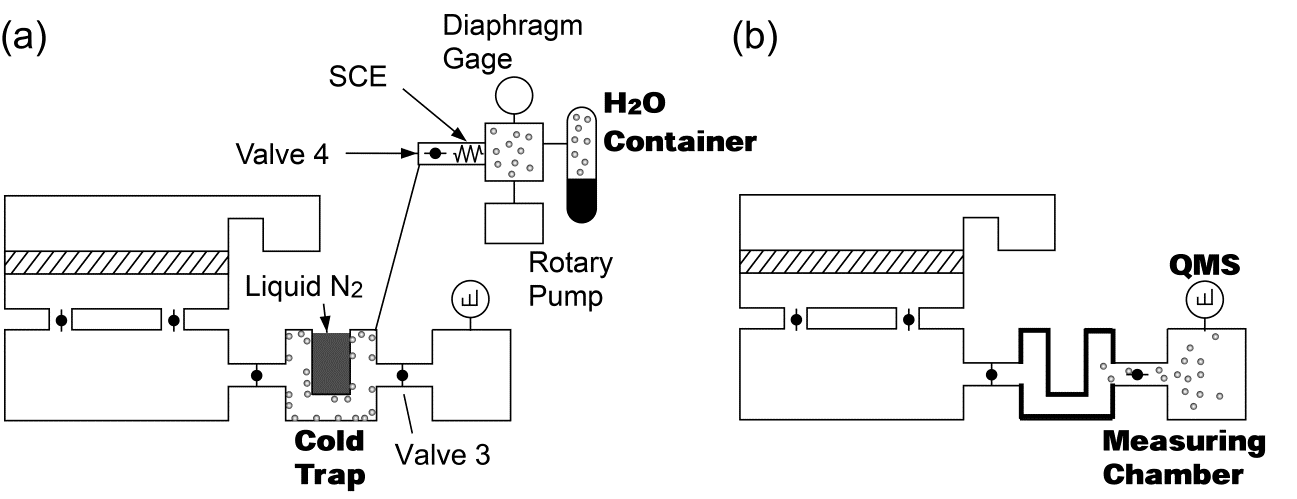


Fig. S3

After opening Valve 4 between the SCE and the cold trap, known flux of H_2_O vapor flowed to the cold trap, where it condensed on the inner surface (Fig. S3(a)).

When a predetermined time (explained later) passed, the Valve 4 was closed and the Valve 3 opened. Then the cold trap was heated to quickly release the H_2_O molecules, which flowed into the measuring chamber with QMS (Fig. S3(b)).

The amount of water vapor flowing through the SCE was determined by multiplying flow rate and time. For the comparison with the measurement of real barrier samples, the amount of water vapor was converted to WVTR, assuming that it had permeated through a Φ60 mm barrier sample in 3 hours, because we usually conduct real measurements under this condition. The amount of the water vapor which flows through the SCE can be adjusted to a certain value by changing both the flow rate and the flow time. We used two SCEs. Their conductance was 1.14 × 10^-9^ and 2.32 × 10^-10^ m^3^ s^-1^. The water vapor flow was adjustable down to 3 × 10^-6^ g s^-1^ with these SCEs, which corresponds to WVTR 10^-3^ g m^-2^ day^-1^. Therefore the flow time was shortened compared to 3h to simulate 10^-6^ g m^-2^ day^-1^ WVTR. For instance, the amount of water vapor which flows at 1 x 10^-6^ g m^-2^ day^-1^ in 3 hours equals to that at 10^-3^ g m^-2^ day^-1^ in 0.003 hours (10.8 sec). The measurements summarized in Table 1 were conducted in this manner. The time required to manually open and close the valves are about 1 s, and the deviation of this handling time was smaller than the deviation of the data corresponding to 10^-5^ ~ 10^-6^ g m^-2^ day^-1^ range. That is the reason why we consider the temperature fluctuation is the main cause of the data deviation.
